# Application of cell-free DNA sequencing in characterization of bloodborne microbes and the study of microbe-disease interactions

**DOI:** 10.7717/peerj.7426

**Published:** 2019-08-06

**Authors:** Kuo-Ping Chiu, Alice L. Yu

**Affiliations:** 1 Genomics Research Center, Academia Sinica, Taipei, Taiwan; 2 Departent of Life Sciences, National Taiwan University, Taipei, Taiwan; 3 Department of Pediatrics, University of California, San Diego, San Diego, United States of America; 4 Institute of Stem Cell and Translational Cancer Research, Chang Gung University, Taipei, Taiwan

**Keywords:** Cell-free DNA, Next-generation sequencing, Microbial species identification, cfDNA sequencing, Microbial cfDNA sequencing

## Abstract

It is an important issue whether microorganisms can live harmoniously with normal cells in the cardiovascular system. The answer to the question will have enormous impact on medical microbiology. To address the issue, it is essential to identify and characterize the bloodborne microbes in an efficient and comprehensive manner. Due to microbial sequence complexity and the composition of significant number of unknown microbial species in the circulatory system, traditional approaches using cell culture, PCR, or microarray are not suitable for the purpose. Recent reports indicate that cell-free DNA (cfDNA) sequencing using next-generation sequencing (NGS) or single-molecule sequencing (SMS), together with bioinformatics approaches, possesses a strong potential enabling us to distinguish microbial species at the nucleotide level. Multiple studies using microbial cfDNA sequencing to identify microbes for septic patients have shown strong agreement with cell culture. Similar approaches have also been applied to reveal previously unidentified microorganisms or to demonstrate the feasibility of comprehensive assessment of bloodborne microorganisms for healthy and/or diseased individuals. SMS using either SMRT (single-molecule real-time) sequencing or Nanopore sequencing are providing new momentum to reinforce this line of investigation. Taken together, microbial cfDNA sequencing provides a novel opportunity allowing us to further understand the involvement of bloodborne microbes in development of diseases. Similar approaches should also be applicable to the study of metagenomics for sufficient and comprehensive analysis of microbial species living in various environments. This article reviews this line of research and discuss the methodological approaches that have been developed, or are likely to be developed in the future, which may have strong potential to facilitate cfDNA- and cfRNA-based studies of cancer and acute/chronic diseases, in the hope that a better understanding of the hidden microbes in the circulatory system will improve diagnosis, prevention and treatment of problematic diseases.

## Introduction

It is important to know whether microorganisms can live harmoniously with normal cells in tissues with close contact with blood circulation, which is equipped with immune surveillance evolved to combat microorganisms. The answer will not only have significant impact on medicine, but also pave the way towards further studies to address key questions, such as how hidden microbes can be tolerated by, or escape from, the immune surveillance. (Here, ‘hidden microbes’ or ‘bloodborne microbes’ are defined as those living within human body and having direct contact with blood circulatory system/ cardiovascular system.) What are the roles played by the hidden microbes in disease development? How microbiota interact with the immune system along the development of a disease? To stratify the interactions between bloodborne microbes and diseases, identification and characterization of the hidden microbes is essential.

Conventional procedure of cell culture followed by laboratory characterization with PCR or microarray has been the gold standard for microbial species identification and characterization. However, it has severe limitations not only in sensitivity and specificity for detecting known species but also being incapable of detecting unknown species. Many microbes in clinical samples are fastidious and either cannot be culturable with conventional approaches or cannot be precisely assigned to specific species. These limitations result in significant false negative in detection and ambiguity in species annotation (Hall & Lyman, 2006; Lamy et al., 2016; Pham & Kim, 2012).

With the advances in sequencing technology, including next-generation sequencing (NGS) and single-molecule sequencing (SMS), and coupled bioinformatics approaches, the above-mentioned issues can now be more effectively addressed at the molecular level using cfDNA sequencing. Recent reports have demonstrated the feasibility of using NGS- and SMS-coupled cell-free DNA sequencing for comprehensive microbiota profiling in bloodstream (De Vlaminck et al., 2015; Grumaz et al., 2016; Hong et al., 2018; Huang et al., 2018; Kowarsky et al., 2017). The same approach is expected to unravel the potential correlation of diseases (e.g., acute or chronic diseases, and cancer) and microbes in blood circulation, especially the involvement of microbes in disease onset or progression. Additionally, similar approaches should be able to enhance the analyses of metagenomics, gastrointestinal microbiota, as well as microbial species in various environments.

Applications of cfDNA sequencing in noninvasive prenatal testing (NIPT) and oncology have been well reviewed (Bianchi & Chiu, 2018; Finotti et al., 2018; Oellerich et al., 2017), except that for bloodborne microbial species identification and characterization. Here, we review its associated technologies, experimental designs and potential applications in microbiology, in hope to facilitate the progress of scientific research in this direction.

### Survey methodology

We surveyed literatures in PubMed and Google Scholar websites using the keywords cell-free DNA, cfDNA and “cell-free DNA” AND “microbial” and included the articles related to our focus. We comprehensively and unbiasedly cover recent articles published before Feb. 2019. This review aims to provide an up-to-date research trend pertaining to the identification of bloodborne microbes associated with cancer or other diseases through cell-free DNA (cfDNA) sequencing.

### Microbes and human diseases

Microbes (e.g., bacteria, fungi and viruses, together with phages that infect prokaryotic cells) form the largest population of biological entities on Earth. Due to constant and intimate contact with humans, their role for the onset and/or development of diseases, including cancer, have long been investigated by academic laboratories, clinics and hospitals worldwide.

Studies have associated numerous types of cancer with viral infections. As absolute pathogens, viruses rely on the biological functions of host cells for replication. This is achieved by internalization of viral genetic material into the host cell followed by manipulation of host’s biological machineries to favor the synthesis of viral nucleic acids and proteins for the assembly of new viral particles. During the process, the host genome may be altered to induce tumorigenesis, such as that shown in Kaposi’s sarcoma induced by HIV (human immunodeficiency virus) or KSHV (Kaposi’s sarcoma herpesvirus) (Mehta et al., 2011; Pyakurel et al., 2007), cervical cancer induced by HPV (human papillomavirus) (Burd, 2003), hepatocarcinoma induced by HBV (hepatitis B virus) (Levrero & Zucman-Rossi, 2016), etc.

On the other hand, bacteria and fungi are free-living microorganisms. Little is known about their roles in cancer or other diseases. However, some unexpected observations were reported. For instance, low levels of pleomorphic bacteria were found in blood circulation of healthy individuals (McLaughlin et al., 2002; Potgieter et al., 2015; Tedeschi et al., 1976). Bacteria were also detected in tumor tissues (Cummins & Tangney, 2013). Since bacteria and fungi, especially the former, may involve in a broad spectrum of host physiology, including metabolism, inflammation, immunity and hematopoiesis, they may play a role in tumorigenesis or cancer development (Roy & Trinchieri, 2017). However, this line of evidence has been elusive and discrete. To stratify the association of microbial cells with diseases, more data are required and the identification and characterization of bloodborne microbes is essential.

### Limitations of cell culture on characterization of bloodborne pathogens

Cell culture using either liquid media or agar plates is a gold standard as well as the mainstream clinical tool for the identification and characterization of infectious agents. Blood agar plates are commonly used in the lab to isolate fastidious bacteria such as *Streptococcus*, *Enterococcus* and *Aerococcus*. Bacterial colonies producing hemolysins capable of lysing red blood cell or hemoglobin are distinguishable based on the color and the degree of transparency in the background (Lorian, 1971).

However, besides the requirement of a long period of waiting (about a week or so) to obtain results and tedious laboratory works, blood culture is hindered by limitation in sensitivity. A literature survey reported that among patients with bloodstream infections, only 5 to 13% of blood cultures turn out to be positive (Lamy et al., 2016). Furthermore, 0.6–6% of positive results were in fact caused by contamination by etiologically irrelevant microbes which are frequently introduced by indwelling vascular access devices (Hall & Lyman, 2006). A much higher ratio of false positive was even estimated independently by another group (Lamy et al., 2016). Even for microbial inhabitants in oral cavity, which acts as a buffer zone between the circulatory system and outside environment, only about 50% of oral microorganisms are culturable (Pham & Kim, 2012; Wade, 2002).

The above-mentioned problems are more or less correlated with the difficulty in culturing microbes growing in natural habitats. It was shown that less than 1% of soil bacteria were culturable in the lab (Pham & Kim, 2012). Since many environmental bacteria may enter human body through wounds or via respiratory or digestive tracks, numerous environmental microbes have the potential to become opportunistic pathogens in hospital (Buonaurio et al., 2002; Huang et al., 2018).

### Cell-free DNA and its potential in diagnostics

Liquid biopsies represent one of the most valuable and readily accessible material for diagnosis and prognosis. For example, plasma cfDNA samples are much more readily available and accessible compared to diseased tissues (e.g., tumor or diseased tissues). A cfDNA sample acts like a genetic reservoir which carries genetic information from all cells within the body (Qin et al., 2016), as DNA fragments are released through apoptosis, necrosis or secretion by virtually all cells within the body, including healthy and diseased cells and microbes (Khier & Lohan, 2018; Kowarsky et al., 2017; Stroun & Anker, 1972).

Although cfDNA samples can be analyzed with PCR or microarray using specific primers or probes to target 16S rRNA or particular genetic sequences in microorganisms, these approaches are not suitable for detecting novel microorganisms. Moreover, primer binding sites or probe-recognition sites may be degraded or fragmented in cfDNA samples.

### NGS-based cfDNA analysis as a robust molecular diagnostic tool

Compared to Sanger Sequencing, NGS conveys a number of advantages including higher speed, high yield and high degree of completeness, but with lower cost, while Sanger Sequencing retains highest accuracy. These advantages have revolutionized biological investigations, making a number of previously untouchable research territories workable (Levy & Myers, 2016), including accelerated human genome sequencing, sequencing of variable regions of antibody-coding genes (Boyd & Crowe Jr, 2016), single-cell sequencing (Chiang et al., 2018; Gawad, Koh & Quake, 2016), and recently developed microbial species sequencing using cfDNA (see below).

By combining the prestigious features of cfDNA with the technological strength of massively parallel sequencing, NGS-based cfDNA sequencing is offering a tremendous momentum to diagnostics. Cell-free DNA sequencing is becoming a robust approach not only for the analysis of disease-associated genetic mutations in cancer and NIPT (Butler et al., 2015; Mehrotra et al., 2018), but also for the identification of fastidious microbes in the circulatory system (Blauwkamp et al., 2019; De Vlaminck et al., 2015; Grumaz et al., 2016; Huang et al., 2018).

Previously cfDNA sequencing focused mainly on the study of human subjects including analysis of mutations in cancer susceptibility genes (Fernandez-Cuesta et al., 2016; Volik et al., 2016), relatively less attention was paid to microbial sequences in cfDNA samples. For microbial DNA analysis, it is practical to just focus on specific sequence markers such as 16S rDNA/rRNA or PCR amplicons (Wade, 2002). Current cfDNA sequencing mainly relies on NGS, while SMS, which is a robust tool for long-read sequencing and which does not necessarily rely on molecular cloning or PCR, is severely hampered by high error rates and random deletions and insertions (Weirather et al., 2017).

### Applications of cell-free DNA in Microbiology

Here, we survey reports published during the past few years, which we believe are most relevant to our subject. While there are other reports more or less relevant to our topic but not included in our discussion, serious readers are encouraged to refer to the literature for additional information.

#### Application in monitoring infection and rejection in lung transplantation patients

It is difficult to use traditional methods to distinguish infection and rejection during organ transplantation, because both have similar clinical symptoms. In their attempt to apply cfDNA sequencing in monitoring infection and rejection during lung transplantation, Vlaminck and colleagues developed a method using single nucleotide polymorphisms (SNPs) to distinguish donor and recipient cfDNA fragments (De Vlaminck et al., 2015). Levels of donor-originated cfDNA were found directly correlated with data from invasive test of rejection. The noninvasive approach avoids invasive tissue biopsies commonly used for detecting rejection. On the other hand, levels of cytomegalovirus-derived cfDNA were found correlated with clinical results of infection.

#### Application in identification of microbes in sepsis

In an attempt to fight septicemia, Grumaz and colleagues reported a cfDNA sequencing approach for detecting the predominant microbial species in bloodstream (Grumaz et al., 2016). Empirically, cfDNA samples were isolated at various time points from three groups including septic patients, surgical patients and normal volunteer controls. Sequencing libraries were constructed and sequenced with HiSeq 2500 to generate 25–30 million 100 bp single-end reads. They first mapped quality reads to human genome assembly hg19. Unmappable reads were then mapped to RefSeq database comprising bacterial, viral and selected fungal genomes. Libraries, including those from septic patients, normal volunteer controls and surgical patients, were normalized and compared.

They were able to obtain results within a period of time shorter than traditional cell culture, which normally requires about 5 days. The identified microbial species were strongly correlated with that obtained by blood cultures. In general, septic patients have highest cfDNA concentrations than surgical patients and volunteer controls. Both Gram-positive and Gram-negative bacteria well matched those identified by corresponding blood cultures. Quantification of microbial species is essential for both cross-species and cross-sample comparisons. The authors employed SIQ values, calculated by complicate formulas, to estimate dynamic microbial cfDNA levels. Some false negatives by cell culture were detected and rescued by cfDNA sequencing.

This work represents a great achievement for sepsis. However, although applicable for septic patients and potentially for other bacteremic cases, mapping with single-end reads of 50–100 bp in length in conjunction with a minimum of 80% identity between read and reference genome may not be stringent enough for the identification of low abundance microorganisms. Additionally, calculation of SIQ seems to be complicate and not very user friendly.

#### Application in discovery of novel bacteria and viruses in bloodstream

By deep sequencing of 1,351 cfDNA samples isolated from 188 patients, Kowarsky and colleagues reported that among a total of 37 billion cfDNA fragments sequenced, 0.45% of reads were unmappable to reference human genome; and among those, only 1% aligned to reference microbiome database composed of about 8,000 species of known bacteria, viruses, fungi and pathogens (Kowarsky et al., 2017). By *de novo* assembly of the non-human sequences, they generated 7,190 contigs. With a series of filtering, testing and merging, they identified 3,761 novel contigs, which were validated not to be artifacts or contaminants. Further analysis indicated that these novel contigs were derived from a vast number of uncharacterized microbes, suggesting that unexpected diversity of novel phages and viruses is present in human body. Thus, microbiome in human body was substantially undersampled previously.

#### Application in identification of bloodborne microbes in healthy females and early-onset breast cancer patients

**Figure 1:**
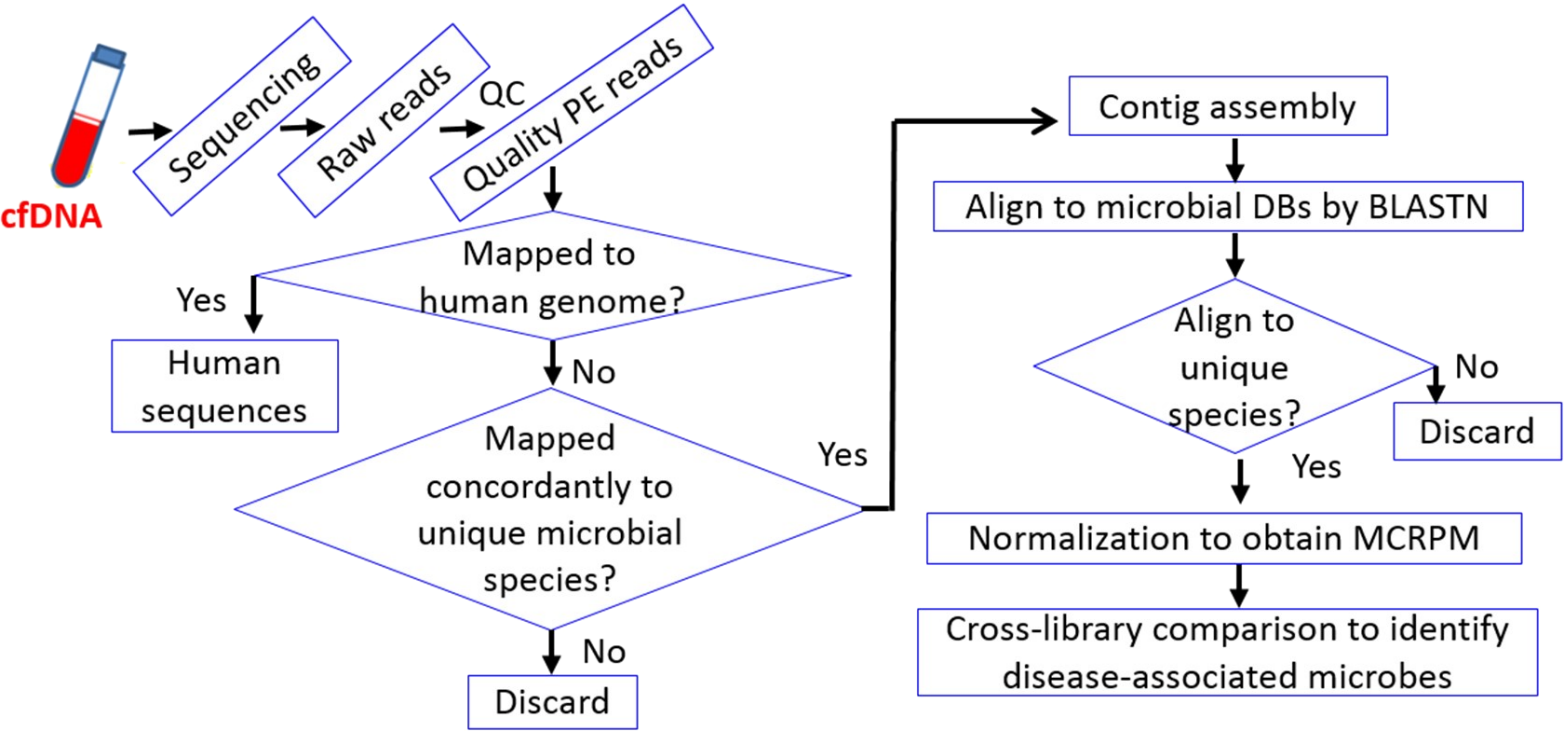
Workflow of cfDNA sequencing andcross-microbial species comparison. The stringent workflow is designed for the identification of rare (as in healthy individuals) and predominant opportunistic pathogens in diseased patients. The stringency relies on initial mapping with Illumina paired-end (PE) reads followed by alignment with contigs assembled from qualified PE reads. Cross-species comparison is based on MCRPM (microbial cfDNA reads per million) values which correlate with the titers of corresponding cfDNA fragments in plasma.

Our recent work further demonstrated the advantages of cfDNA sequencing in microbial species identification for both healthy individuals and early-onset breast cancer (EOBC) patients (Huang et al., 2018). The work relies on a stringent pipeline for mapping and alignment of NGS paired end (PE) reads and contigs, respectively, to identify microbial inhabitants (Fig. 1). The relative levels of microbe-associated cfDNA were compared under MCRPM (microbial cfDNA reads per million) basis after normalization. Healthy individuals were found to have similar bacterial profiles. Patient with a profile similar to that of healthy individuals indicated a fair recovery, while patients with advanced EOBC were found to be infected by opportunistic pathogens. Microbial profiles can thus be associated with health conditions. Since spreading of microbes is strongly influenced by the environment, it is not possible to directly correlate a particular disease, or its clinical status, with particular microbes. The approach paves the way toward a better understanding of the role played by microbes in development and/or prognosis of cancer, and possibly chronic diseases as well.

Microbial genomes are much smaller (most in the range of a few Mb) than the human genome (∼3 Gb for haploid cells). In order to obtain carbon and energy sources, certain genomic regains are able to alter quickly through mutations or horizontal gene transfer. Together with other reasons, genomes of related microbes have high degree of sequence similarity, making species identification by mapping or alignment a great challenge, especially when cfDNA-derived paired-end reads or single-end reads are used. Instead, contig-based alignment is supposed to be a better choice for improving accuracy. Results indicated that cfDNA-identified microbial profile can function as a prognostic indicator or reference.

#### Validation of microbial cfDNA sequencing test showed a strong agreement with blood culture

Recently, an analytical study was conducted by Blauwkamp et al. to validate microbial cfDNA sequencing test (Blauwkamp et al., 2019). In the study, factors that may affect accuracy, precision or that may introduce bias were examined, and results were validated with *in silico* simulation, to evaluate the reliability and robustness of microbial cfDNA sequencing for assessing clinically relevant microbes or eukaryotic parasites (1,250 predefined). Results were then compared to cell culture. It is noteworthy that microbe diagnosis for acute infections (e.g., sepsis) may focus on major microbial species in the bloodstream, while mild infections or chronic diseases may require more comprehensive assessment to identify all potential pathogens in patients.

To ensure reliability, the authors used 13 microorganisms as reference. Various cfDNA concentrations were prepared and calculated, in microbe-specific cfDNA per microliter of plasma (MMP), so that factors that may affect reliability (e.g., limit of detection, limit of quantitation, accuracy, precision and linearity, etc.) can be validated at different concentrations (i.e., low, medium and high). The authors also employed fragmented DNA samples from two pairs of closely related bacteria (i.e., *E. coli/S. flexneri* and *S. aureus/S. epidermidis*) to ensure similar microbial species can be properly discriminated.

Results showed 93.7% agreement with blood culture for a cohort of 350 patients with sepsis alert and 62 (37.3%) out of 166 samples with no sepsis aetiology were found to contain microbial cfDNA. Genetic factors such as GC content, genome size, and sequence similarity (when difference >3%) seemed to have no significant effect on the results.

A strong positive agreement for patients with sepsis alert indicated that microbial cfDNA sequencing is a reliable approach for the assessment of microbial species in blood circulation.

### Analysis of cfDNA in non-blood body fluids

Besides blood plasma, other liquid biopsies also deserve a careful scrutiny. These include saliva, urine, tears, lymph, vaginal discharge, semen, cerebrospinal fluid, etc. Reports have indicated a strong potential of non-blood cfDNA in diagnostics of diseases, especially for local neoplasms (Peng et al., 2017) and infections (Burnham et al., 2018).

Saliva consists of a large spectrum of informative materials, including cfDNA and exosome (Hyun et al., 2018), mRNA, and proteins of immunological, toxicological, hormonal and therapeutic values. Applications of salivary fluid cover not only the genomics (including cancer genomics), transcriptomic and proteomics of the individual, but also the microbiota in the oral cavity. Reports have demonstrated the feasibility of using saliva in oral cancer diagnosis (Hyun et al., 2018), and the study of EGFR mutation for lung cancer patients (Wei et al., 2014). Lymphatic fluid has close contact with neurons and thus may be used for the detection of neuronal pathogens. Vaginal discharge and semen samples can be useful specimen for testing pathogens associated with sexually transmitted diseases (e.g., gonorrhea and syphilis) or cancer (e.g., cervical cancer) such as *Neisseria gonorrhoeae* that causes gonorrhea, spirochete *Treponema pallidum* that cause syphilis and HPV that causes cervical cancer. These body fluids have close contact with certain types of tissues mediated with particular pathogenic infections and thus may have collected desired genetic material or microbial cells for clinical application.

Urine has also been reported to be useful for the study of cancer, such as colorectal and prostate cancers, and the *BRAF* V600e mutations in Erdheim-Chester syndrome. The cell-free DNA fragments in urine are known to be between 150–250 bp in size. However, high false negative rate was reported due to limited volume or lack of advanced technology.

Urinary cfDNA was reported to be a versatile specimen for monitoring urinary tract infection (UTI) (Burnham et al., 2018). UTI is a common problem for kidney transplant recipients (Abbott et al., 2004), ∼20% of the recipients are affected by UTI during the first year after transplantation (Ariza-Heredia et al., 2014), and over 50% are affected within the first three years (Chuang, Parikh & Langone, 2005). The authors analyzed 141 urinary cfDNA samples of a cohort composed of 82 kidney transplant recipients. Results indicated that a single urinary cfDNA sample is informative enough to provide multiple pieces of information including microbial composition and dynamics, kidney allograft injury, host response to UTI, antimicrobial susceptibility and bacterial growth dynamics.

Since cfDNA fragments are predominantly from normal cells, finding those released from diseased cells is like finding a needle in a haystack. To get around the problem, cfDNA analysis normally requires advanced methodologies such as PCR and NGS. Moreover, selection of body fluid in close proximity to diseased tissue for cfDNA analysis is also one way to improve the efficacy (Burnham et al., 2018; Lin et al., 2017; Markopoulos, Michailidou & Tzimagiorgis, 2010; Salvi et al., 2015).

### Limitations of cfDNA sequencing

Cell-free DNA sequencing has limitations in a number of aspects. There are technical issues. Up to date, microbial cfDNAs are analyzed as a small constituent of a global cfDNA genetic information repertoire. Under such circumstance, human sequences are first identified by mapping/aligning of quality reads against a human genome assembly and then removed, leaving microbial sequences for further analysis. The procedure does not involve enrichment for microbial sequences. Large amount of human cfDNAs, much more than that derived from microbes, is present in the initial sequencing libraries, making the analysis tedious and costly.

In theory, enrichment of microbial cfDNA fragments prior to sequencing library construction may sound like a reasonable alternative. However, enrichment is frequently accompanied by side effects. First of all, enrichment targets only desired microbial sequences and ignore not only human sequences, but all novel microbial sequences, which may also associate with the disease of interest. Moreover, cfDNAs are normally of low quantity, enrichment may cause significant loss of desired fragments from the repertoire and thus introduce bias to the analysis.

In terms of sequencing, recent advances in SMS provide a new momentum to microbial cfDNA sequencing. However, there are two evident drawbacks that limit the application of SMS in this field, i.e., high (5–20%) sequencing errors and long read length (Sedlazeck et al., 2018). As a pocket-sized sequencer commercialized by Oxford Nanopore Technologies (ONT), MinION has demonstrated a number of advantages over any other sequencers. These include long read length, direct RNA sequencing, high portability (suitable for field testing) and low cost (Greninger et al., 2015; Kafetzopoulou et al., 2018). Direct RNA sequencing permits direct sequencing of RNA microbial genomes. However, to employ SMS long read sequencing to study short-length cfDNAs, concatemerization of short cfDNA fragments prior to sequencing library construction seems inevitable, and further improvements of protocol and experimental procedure are also needed.

There are also intrinsic issues affecting the accuracy and reliability. As reviewed by Khier and Lohan, while cfDNA fragments travel across different tissues and present in various body fluids, their levels are influenced by physiological barriers (Khier & Lohan, 2018). We assume the distribution of microbial cfDNA to be influenced in similar manner. Moreover, biophysical barrier resulted from the presence (Gram-positive) or absence (Gram-negative) of cell wall may also affect the kinetics of microbial cfDNA, although not yet empirically demonstrated. Fortunately, due to the short half-life of cfDNA, which ranges between seconds or minutes (Gosse et al., 1965; Khier & Lohan, 2018), the presence of a cfDNA sequence remains to be a trustworthy indicator for the presence of its corresponding (live) cells, especially when a cutoff threshold (e.g., MCRPM) is applied (Huang et al., 2018).

Accuracy is crucially important for microbial cfDNA analysis. Since the scope of microbial databases determines the scope of microbial species that can be assigned, the degree of completeness in microbial sequence collection is critically important. As such microbial databases need to be updated constantly with newly discovered microbial sequences. Given the fact that the majority of microbial species remains undiscovered, constant updating the databases is certainly a great challenge.

### Future prospective

Certain chronic diseases may result in substantial alteration in physiology, which may be reflected by microbial profile and detected by cfDNA sequencing. Diabetes and Alzheimer’s disease are good examples. In theory, elevated glucose level in diabetic patients may selectively provoke an outgrowth of certain types of microbes yet to be identified. These microbes may themselves participate in the progression of diabetes and/or patients’ health conditions. Probing the microbes in diabetic patients may provide important information for treatment. Alzheimer’s disease represents a good example for combinatorial analysis of a cfDNA sample. Progressive neuronal cell death along the progression of the disease would release increasing amount of related cfDNA into the bloodstream or other body fluids. Cell-free DNA sequencing should be able to provide real time information to expedite the treatment of the disease. Moreover, a recent study has associated herpesviruses HHV-6A and HHV-7 with the development of Alzheimer’s disease (Readhead et al., 2018). Cell-free DNA sequencing may allow us to correlate herpesviral cfDNA titer with the progression of the clinical symptom. Furthermore, since Alzheimer’s disease is characterized as a disease with accumulation of beta-amyloid peptide, it would be attempting to correlate herpesviral profile with beta-amyloid profile. Other diseases, such as Parkinson’s disease and gout, may also be good putative candidates to be studied by cfDNA sequencing.

Once the major microbes are identified from cfDNA sequencing, a series of characterization or antimicrobial susceptibility testing can be performed. The results will be able to help doctors to determine what drugs to use for treating the patient.

## Conclusions

As one of the major living entities on Earth, microorganisms play a key role in human diseases, and identification/characterization of disease-associated microorganisms is crucially important for treatment. Previous approaches using cell culture, PCR and other conventional clinical methods are hindered not only by limited sensitivity and specificity, but also by accessibility, when bloodborne microbes are of concern. Owing to the robustness of next-generation sequencing and single-molecule sequencing, direct cfDNA analysis represents a superior approach allowing us to probe the hidden microbes without going through the conventional methods. Increasing number of reports have been showing promising results to support this notion.

However, there are still things to be done to expedite the application of cfDNA sequencing in the study of hidden microbes (please refer to the ‘Limitations of cfDNA sequencing’ section). Besides, after microbiome profiling, it would be useful to track down the source tissues for microbes of interest. For tracking down the source tissues where the microbes reside, additional information such as cfDNA-associated tissue-specific markers or others, if present, may be needed, but has yet to be further developed. There is no doubt that this line of research will facilitate our understanding of how microorganisms participate in the onset and development of diseases, and thus may provide novel approaches for diagnosis and early detection of diseases.

## Additional Information and Declarations

### Data Availability

The following information was supplied regarding data availability: This is a review article; there is no raw data.
